# Effects of pyrroloquinoline quinone and imidazole pyrroloquinoline on biological activities and neural functions

**DOI:** 10.1016/j.heliyon.2020.e03240

**Published:** 2020-01-29

**Authors:** Yasue Yamada, Kazuya Nishii, Koji Kuwata, Masashi Nakamichi, Kei Nakanishi, Atsushi Sugimoto, Kazuto Ikemoto

**Affiliations:** a Department of Biotechnology and Chemistry, Faculty of Engineering, Kindai University, Higashi-Hiroshima, Hiroshima, 739-2116, Japan; b Niigata Research Laboratory, Mitsubishi Gas Chemical Company, Inc., Niigata, 950-3112, Japan

**Keywords:** Food technology, Food quality, Food analysis, Food chemistry, Nutrition, Cytochrome c oxidase subunit IV isoform I, Cognitive function., Pyrroloquinoline quinone, Mitochondriogensis, Imidazol pyrroloquinoline

## Abstract

Pyrroloquinoline quinone (PQQ) is contained in fruits and vegetables and in human breast milk. It has been reported that PQQ has high reactivity and changes to an imidazole structure (imidazole pyrroloquinoline) by a reaction with an amino acid at a high ratio in nature. A comparative study was conducted to clarify physiological effects including neuroprotective effects, growth-promoting effect, antioxidative effects and a stimulatory effect on mitochondriogensis of PQQ and imidazole pyrroloquinoline (IPQ) using a human neuroblastoma cell line and a hepatocellular carcinoma cell line. We also compared the expression levels of human cytochrome c oxidase subunit IV isoform Ⅰ (COX4/1), which is an index of the amount of mitochondria in the cells that had been exposed to PQQ, PQQH_2_ and IPQ. The results of the comparison showed that IPQ had almost the same biological activities as those of PQQ except for anti-oxidative activity. It was also shown that PQQ and IPQ improve the memory learning ability of aged mice and that BioPQQ® improves brain function in the language field in humans.

## 1 Introduction

**Figure 1:**
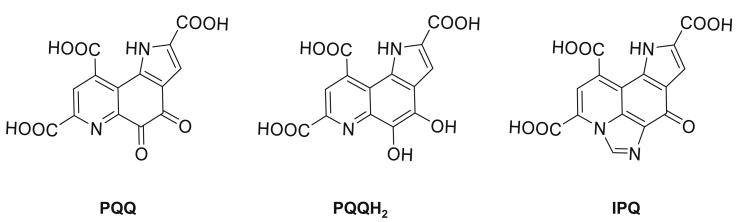
Comparison of structures of PQQ, PQQH_2_ and IPQ.

Pyrroloquinoline quinone (PQQ) was discovered in 1979 from gram-negative bacteria [1, 2, 3, 4] and it is known to be contained in fruits and vegetables such as kiwi fruit and green peppers and in human breast milk [5, 6, 7]. In 2016, PQQ was reported to be a new mammalian lactate dehydrogenase (LDH) coenzyme [8]. PQQ has diverse effects including an antioxidant effect, a cell growth-promoting effect, and a stimulatory effect on mitochondriogensis [9, 10, 11, 12, 13, 14]. PQQNa_2_ is the most common source of PQQ and is used as a functional food. It was reported that the antioxidant effect of PQQ depends on the reductivity of the quinone moiety [15]. However, the mechanisms of other actions of PQQNa_2_ (oxidized PQQ) and reduced PQQ (PQQH_2_) are still unclear. PQQ has a very high reactivity and readily reacts with amino group-containing substances, and it has been reported to change to imidazol pyrroloquinoline (IPQ) by forming an imidazole skeleton (Figure 1). In the presence of glycine, 100% of PQQ is converted to IPQ [7, 16]. It may react with an amino group-containing substance to form IPQ *in vivo*. Some biological activities (cell growth promoting activity and radical scavenging activity) of IPQ have been reported [17, 18, 19]. However, biological activities of IPQ have still not been investigated in detail. We investigated whether IPQ functions *in vivo* by using the human neuroblastoma cell line SK-N-SH and the human liver hepatocellular carcinoma cell line HepG2. Hydrogen peroxide (H_2_O_2_) is one type of reactive oxygen species (ROS) [20]. Neurons are vulnerable to oxidative stress. Parkinson's disease (PD) is one of most common neurodegenerative diseases with progressive neurodegeneration of the nigrostriatal pathway. The dopamine analog 6-hydroxydopamine (6-OHDA) has been reported to induce PD in animal experiments [21]. 6-OHDA is a neurotoxin that acts specifically on nerve cells and is taken up by dopaminergic neurons. It was shown by using human neuroblastoma SH-SY5Y cells that 15 μM of PQQ has a protective effect against 6-OHDA [22]. It is thought that ROS and endoplasmic reticulum stress (ERS) are involved in the toxicity of 6-OHDA. In this study, we investigated various protective effects of PQQ, PQQH_2_, and IPQ in SK-N-SH cells against 6-OHDA, H_2_O_2_, and tapsigargin (TG). TG induces ERS by inversely inhibiting Ca^2+^-ATPase (SERCA) on the endoplasmic reticulum membrane, causing cell death [23].

It has been shown that mice and rats fed a diet lacking PQQ have a reduced amount of mitochondria [13, 24]. By using mouse Hepa1-6 cells, it was shown that PQQ stimulated mitochondorial biogenesis by phosphorylation of CREB (cAMP responsive element-binding protein) and by increasing the mRNA and protein expression levels of PGC1α (peroxisome proliferator-activated receptorγco-activator-1α), which is transcription factor related to mitochondriogenesis [14]. To examine the stimulation of mitochondriogenesis in human cells by PQQ, PQQH_2_, and IPQ, the expression levels of human COX 4/1 (human cytochrome c oxidase subunit IV isoform I) and PGC1α were examined.

It was reported that PQQ enhanced the production of nerve growth factor (NGF) in the mouse fibroblast cell line LM [12, 25]. By using a Morris water maze apparatus, it was shown that PQQ had an effect on cognitive function in aged rats [26]. PQQ may be used as an ingredient in food that is effective for improving brain cognitive function. In order to examine the memory improving effects of PQQ and IPQ, a step-through type passive avoidance test was conducted after feeding PQQ and IPQ for 7 days to aged mice. The Montreal Cognitive Assessment (MoCA) is a simple and sensitive screening assessment for mild cognitive impairment (MCI). MoCA covers the major cognitive domains. To evaluate the effects of PQQ on human brain cognitive function, MoCA was performed in forty healthy men and women aged 50–71 years [27].

## 2 Materials and methods

### 2.1 Samples and materials

Rotenone3-(4,5-dimethylthiazol-2-yl)-2,5-diphenyltetrazolium bromide (MTT), glutamine, ethylenediaminetetraacetic acid (EDTA), and trypsin were purchased from Sigma-Aldrich (St Louis, MO). Modified Eagle's medium α (MEMα) and MEM were purchased from GIBCO/Thermo Fisher Scientific (Waltham, MA). Fetal calf serum (FCS) was purchased from Biosciences SIGMA/Sigma-Aldrich. The PQQ source was PQQNa_2_ (BioPQQ®; Mitsubishi Gas Chemical Co. Inc., Japan). The reduced form of PQQ (PQQH_2_, free form) was made from a reaction of PQQNa_2_ and excess ascorbic acid. Pure PQQH_2_ was precipitated from the water solution of the reaction mixture. The precipitate of pure PQQH_2_ is a proton adduct (free form). IPQ (IPQNa_3_) was made from a reaction of PQQNa_2_ and glycine, and it was purified by recrystallization as previously reported [28]. The data for IPQNa_3_ are: 1H-NMR (D2O, TSP): 7.24, 8.20, 9.35 ppm; 13C-NMR (D2O, TSP): 112.45, 118.56, 123.79, 128.59, 130.81, 133.17, 134.10, 135.05, 135.09, 136.94, 138.66, 169.63, 170.90, 174.54, 178.89 ppm; MS (ESI positive): 342.0 (M+1 = IPQ+1). A sodium ion meter (LAQUA twin B–722Na+; Horiba Scientific, Kyoto, Japan) was used for sodium analysis. These data indicated the presence of trisodium salt.

### 2.2 Cell culture

SK-N-SH cells and HepG2 cells were purchased from Institute of Physical and Chemical Research (Japan) (IPCR, RIKEN) and were grown in MEMα and MEM supplemented with 2 mM glutamine and 10% heat-inactivated FCS, respectively. The cells were maintained at 37 °C under 5% CO_2_/95% humidity, and the medium was changed three times a week. SK-N-SH cells and HepG2 cells were incubated with various concentrations of the materials for 10 min. SK-N-SH cells were incubated in the presence of 6-OHDA for 24 h in a CO_2_ incubator or in the presence of H_2_O_2_ for 10 min with or without PQQ, PQQH_2_, or IPQ at room temperature. After incubation, cell viability rates were determined measured by an MTT assay.

### 2.3 MTT assay

Cell viability was analyzed using the conventional MTT reduction assay. The cells were incubated in the presence of 6-OHDA for 24 h in a CO_2_ incubator, in the presence of H_2_O_2_ for 10 min at room temperature, and in the presence of TG for 24 h in a CO_2_ incubator. After incubation, cell viability rates were determined by an MTT assay. Cells were incubated with MTT (5 mg/ml) at 37 °C for 4 h. After incubation, the medium was removed, and the cells were dissolved in an acid-dissolving solution in which 5% sodium dodecyl sulfate (SDS) in 0.01 M HCl and 0.02 M HCl-isopropanol had been mixed in equal amounts. Absorbance of the formazan reduction product was measured at 570 nm in a plate reader.

### 2.4 Animal experiments

Male C57BL/6 mice at 48 weeks of age (Charles River Japan, Yokohama, Japan) were maintained under controlled conditions (ambient temperature, 22 °C ± 2 °C; 12 h light/dark cycle, lights on from 0:00 am to 12:00 pm). The animals had free access to food and water. All animals received humane care as outlined in the Guide for the Care and Use of Laboratory Animals established by Kindai University Animal Care Committee (permit number: KAEN-23-001).

### 2.5 Expression of COX4/1 and PGC1α

Total mRNAs were extracted from SK-N-SH cells that had been cultured for 24 h after the addition of 100 nM of PQQ, PQQH_2_, or IPQ. Total mRNAs were also extracted from HepG2 cells that had been cultured for 24 h after the addition of 1 μM of PQQ, PQQH_2_, or IPQ. Total RNAs were isolated from cultured cells with SV Total RNA Isolation System (Promega, Fitchburg, WI). The total RNAs (3–5 μg) were transcribed into cDNA using an iScriptTM cDNA synthesis kit (Bio-Rad, Hercules, CA). Real-time quantitative polymerase chain reactions were performed in an Eco real-time PCR system (Illumia, San Diego, CA) in combination with TaqMan probes (Applied Biosystems) for COX4/1 and PGC1 α. The relative quantities of COX4/1 and PGC1α transcripts were normalized to the level of GAPDH message.

### 2.6 Step-through passive avoidance test

Saline was intraperitoneally (i.p.) administered for 7 days before the tests as a control (n = 8 for each experiment). PQQNa_2_ (5.0 mg/kg BW) or IPQNa_3_ (5.0 mg/kg BW) were i.p. administered for 7 days before the tests (n = 8). All reagents were dissolved in sterilized saline. After individual trials, the apparatus was carefully cleaned with a wet paper towel (soaked in a mixture of ethanol and water) to remove any residues or odors. The passive avoidance test was conducted in a two-compartment box with one bright compartment and one dark compartment connected by a sliding door. On the training day, the mouse was allowed to acclimatize for 60 s in the bright compartment of the device. Thereafter, each mouse was again placed in the bright chamber. The door was opened, and the time taken for the animal to enter the dark compartment was recorded as the initial latency (IL). When the mouse moved to the dark side of the device, foot impact (0.1 mA, 1.0 s) was generated through the grid floor. On the test day (24 h after the training test), each mouse was placed in the bright compartment and the latency (step through latency, STL) to enter the dark compartment was recorded without electrical foot shock. If the mouse did not enter the dark side within 300 s, it was removed from the device and given a latency of 300 s for the test. STL was used as a measure of memory retention [29]. All experiments were performed with conscious mice unless otherwise noted. The experimental protocol was approved by Kindai University's Committee for the Care and Use of Laboratory Animals ((permit number: KAEN-23-001)).

### 2.7 MoCA test

A double-blind, placebo-controlled, parallel-grouped randomized clinical study was conducted in healthy middle-aged and elderly subjects. Forty healthy men and women aged 50–71 years (14 men and 26 women) were assigned to a PQQ-containing food intake group (BioPQQ® at 20 mg per day) and a placebo food intake group. The dose of PQQ disodium salt was based on our previous study [27, 30]. To achieve a uniform test score, each group was randomly allocated by age and gender before ingestion of the test food. Placebo capsules each contained 187 mg of starch, 8.8 mg of caramel coloring, 11 mg of calcium stearate and 13.2 mg of starch hydrolysis material. PQQ capsules each contained 10 mg of BioPQQ®, 150.5 mg of starch, 7.5 mg of calcium stearate, and 82 mg of starch hydrolysis material. Each subject ingested 2 capsules after breakfast everyday for 12 weeks. The MoCA test consists of 30 points (Visuospatial: 5, Naming: 3, Attention: 6, Language: 3, Abstraction: 2, Delayed recall: 5, Orientation: 6). The tests were conducted by CX Medical Japan Co., Ltd., which was entrusted by Mitsubishi Gas Chemical Company, Limited. The testing facility was Kagawa Nutrition University Nutrition Clinic. The tests were conducted from November 19, 2016 to March 24, 2017. The test was approved by the Ethics Review Committee of Kagawa Nutrition University. This study was also conducted under an ethical arrangement in accordance with the Helsinki Declaration (revised by the Fortaleza General Assembly in 2013).

### 2.8 Statistical analysis

Data are expressed as means ± SEM. Statistical analysis were performed by using Dunnett's test or Student's t-test. A p-value of 0.05 or less was considered statistically significant.

## 3 Results

### 3.1 Comparison of the cytotoxicities of PQQ, PQQH_2_ and IPQ

To investigate the differences between the cytotoxicities of PQQ, PQQH_2_ and IPQ (Figure 1), SK-N-SH and HepG2 cells were incubated with various concentrations of PQQ, PQQH_2_ and IPQ for 24 h. The IC_50_ values for PQQ in SK-N-SH and HepG2 cells were calculated to be 0.17 mM and 0.43 mM, respectively, and those for PQQH_2_ were calculated to be 0.25 mM and 0.19 mM, respectively (Table 1, Figure 1). The IC_50_ values for IPQ in SK-N-SH and HepG2 cells were calculated to be 3.5 mM and 1.15 mM, respectively (Table 1). The viabilities of the cells in wells not containing PQQ, PQQH_2_ or IPQ were considered to be 100% and were used as a reference control. IPQ showed lower cytotoxicity than that of PQQ and PQQH_2_ in SK-N-SH and HepG2 cells.

**Table 1:** Comparison of the cytotoxicities of PQQ, PQQH_2_ and IPQ.

Substrate	Cell line	IC_50_ (mM)
PQQ	SK-N-SH	0.17
	HepG2	0.43
PQQH_2_	SK-N-SH	0.25
	HepG2	0.19
IPQ	SK-N-SH	3.50
	HepG2	1.15

### 3.2 Growth promoting effects of PQQ, PQQH_2_ and IPQ

**Figure 2:**
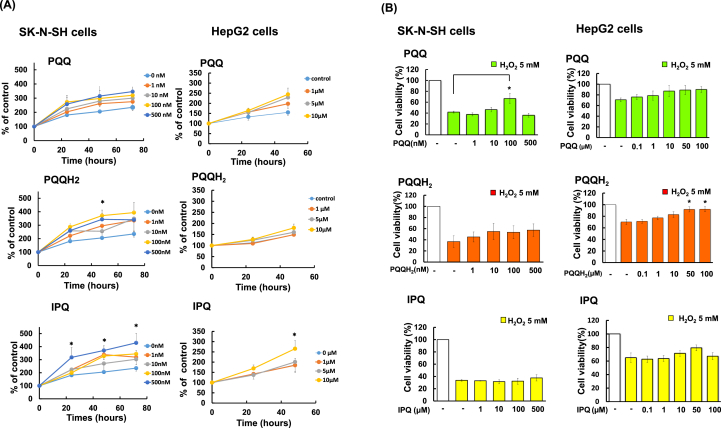
Comparison of the effects of PQQ, PQQH_2_ and IPQ on cell proliferation and their protective effects against cell death caused by H_2_O_2_ in SK-N-SH cells and HepG2 cells. (A) To determine the effects of PQQ, PQQH_2_ and IPQ on cell proliferation, SK-N-SH cells and HepG2 cells were incubated with various concentrations of PQQ and IPQ for 72h and 48 h, respectively. After incubation, cell viability was determined by an MTT assay. Each value is the mean ± SEM (n = 3–5). *p < 0.05 vs control by Dunnett's test. (B) Protective effects of PQQ, PQQH_2_ and IPQ against cell death caused by H_2_O_2_. SK-N-SH cells and HepG2 cells were exposed to H_2_O_2_ (5 mM) in the presence or absence of PQQ and IPQ for 10 min. After incubation, cell viability was determined by an MTT assay. Cell viability without H_2_O_2_ was considered to be 100%. Each value is the mean ± SEM (n = 3–5). **p < 0.01, *p < 0.05 vs 5 mM H_2_O_2._ by Dunnett's test.

To examine the effects of PQQ, PQQH_2_ and IPQ on proliferation of cells, SK-N-SH and HepG2 cells were cultured with various concentrations of PQQ, PQQH_2_ and IPQ for 72 h and 48 h, respectively. Cell viability was measured using an MTT assay. PQQ and IPQ showed cell proliferative effects in a concentration-dependent manner, though PQQH_2_ had no effect on HepG2 cells (Figure 2A). It was shown that SK-N-H cells (1–500 nM), which are neural cells, are more susceptible to PQQ and IPQ than are HepG2 cells derived from the liver (1–10 μM). The results indicate that the reactivity of PQQ and IPQ varies depending on the tissues or cells.

### 3.3 Protective effects of PQQ, PQQH_2_ and IPQ against cell death caused by H_2_O_2_ in SK-N-SH cells and HepG2 cells

The MTT assay was performed to examine the protective effects of PQQ, PQQH_2_ and IPQ on SK-N-SH and HepG2 cell death by incubation of the cells with H_2_O_2_ for 10 min. As shown in Figure 2B, PQQ at 100 nM showed a significant protective effect against SK-N-SH cell death caused by H_2_O_2_. PQQH_2_ showed a weak protective effect on the cells. IPQ at 1–500 μM showed no protective effect. PQQ showed a weak protective effect on HepG2 cells. PQQH_2_ at 50 μM and 100 μM showed a significant protective effect on cells. IPQ showed a weak protective effect on the cells. The results showed that PQQ has higher anti-oxidative activity than that of IPQ. These results indicate that the anti-oxidative activity of PQQ and IPQ varies depending on the tissues or cells.

### 3.4 Comparison of the protective effects of PQQ, PQQH_2_ and IPQ against death of SK-N-SH cells induced by 6-OHDA and TG

**Figure 3:**
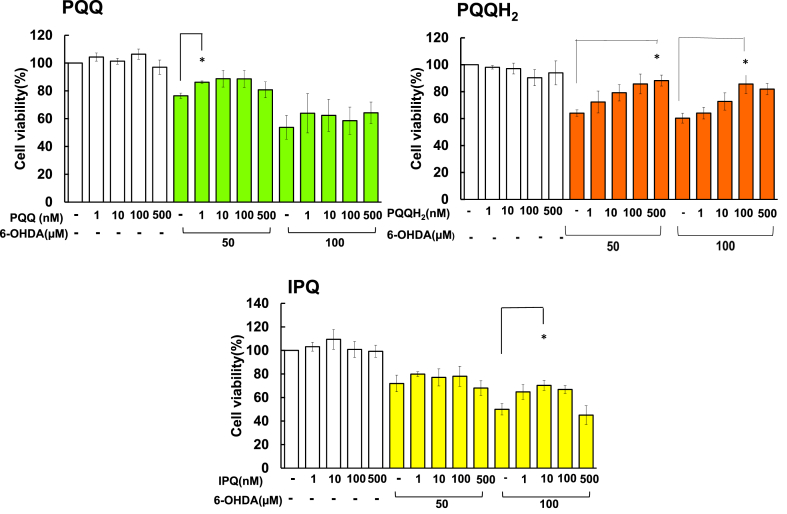
Comparison of the protective effects of PQQ, PQQH_2_ and IPQ against cell death induced by 6-OHDA. SK-N-SH cells were exposed to 6-OHDA (0, 50 μM and 100 μM) for 24 h in the presence of PQQ, PQQH_2_, or IPQ. After treatment, cell viability was determined by an MTT assay. Values (means ± SEM, n = 3–5) are expressed as percentages relative to untreated cells. **p < 0.01, *p < 0.05 vs control by Dunnett's test.

To compare the abilities of PQQ, PQQH_2_ and IPQ to protect against 6-OHDA-induced neurotoxicity, SK-N-SH cells were exposed to 6-OHDA (0, 50 μM and 100 μM) for 24 h in the presence of PQQ, PQQH_2_ or IPQ (Figure 3). They prevented 6-OHDA-induced cell death at concentrations of 1 nM–100 nM. These results indicate that PQQ, PQQH_2_ and IPQ exert protective effects against 6-OHDA-induced neurotoxicity at low concentrations. After treatment, cell viability was measured by the MTT assay. Values (means ± SEM, n = 3–5) are expressed as percentages relative to untreated cells. PQQ, PQQH_2_ and IPQ did not show protective effects against cell death caused by TG (data not shown).

### 3.5 Expression of COX4/1 and PGC1a after incubation with PQQ, PQQH_2_ and IPQ

**Figure 4:**
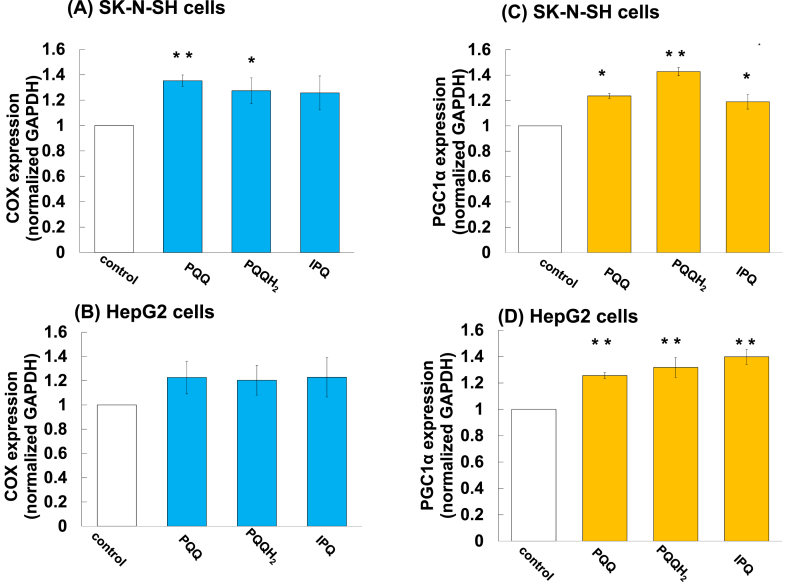
Expression of COX4/1 and PGC1α in SK-N-SH cells and HepG2 cells after incubation of the cells with PQQ, PQQH_2_ and IPQ. (A) COX4/1 expression in SK-N-SH cells, (B) COX4/1 expression in HepG2 cells (C) PGC1α expression in SK-N-SH cells, (D) PGC1α expression in HepG2 cells. Total mRNAs were extracted from SK-N-SH cells and HepG2 cells that had been cultured for 24 h after the addition PQQ, PQQH_2_ and IPQ. One hundred nM and 1 μM of PQQ, PQQH_2_ or IPQ were added to SK-N-SH cells and HepG2 cells, respectively. Total RNAs were isolated from cultured cells. Total RNAs (3–5 μg) were transcribed into cDNA by real-time quantitative polymerase chain reactions with TaqMan probes for COX4/1 and PGC1α. The relative quantities of COX4/1 and PGC1α transcripts were normalized to the level of GAPDH message. Each value is the mean ± SEM (n = 3–5). ***p < 0.001, **p < 0.01, *p < 0.05 vs control by Dunnett's test.

It has been reported that PQQ is involved in energy production and in an increase in the amount of mitochondria. RT-PCR was performed using the TaqMan probe in order to determine the increase in the amount of mitochondria. As shown in Figure 4, the expression level of COX4/1 was significantly increased in the presence of 100 nM PQQ, PQQH_2_ or IPQ in SK-N-SH cells. In the presence of 1 μM PQQ, PQQH_2_ or IPQ, the expression level of COX4/1 showed a tendency to increase in HepG2 cells. The expression level of PGC1α was also significantly increased in SK-N-SH cells and HepG2 cells in the presence of PQQ, PQQH_2_ and IPQ. GAPDH, which was considered to be present in the same amount in all cells, was used as an internal standard substance. PQQ, PQQH_2_ and IPQ at the concentration of 100 nM increased the expression levels of COX4/1 and PGC1α in SK-N-SH cells. It was revealed that PQQ and IPQ increase the amount of mitochondria also in the human nervous system. Potentiation of mitochondrial production might ultimately promote growth of neural cells and liver cells.

### 3.6 Effects of PQQ and IPQ on learning memory

**Figure 5:**
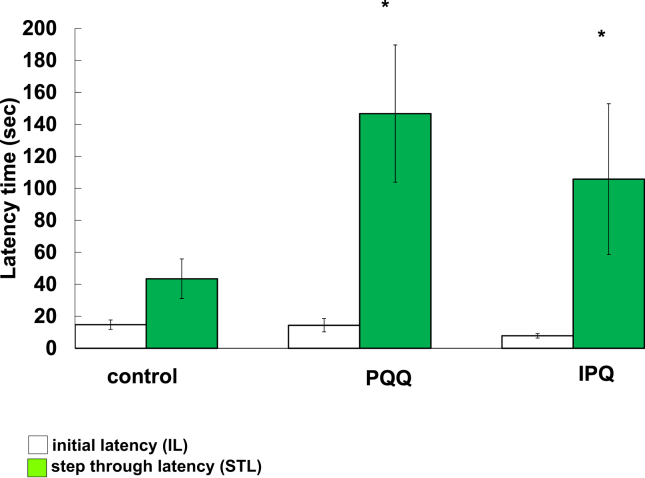
Step though latency of the passive avoidance task for mice administered PQQ and IPQ. Saline was intraperitoneally (i.p.) administered for 10 days before the tests as a control (n = 8 for each experiment). PQQNa_2_ (5.0 mg/kg BW) and IPQNa_3_ (5.0 mg/kg BW) were i.p. administered for 7 days before the tests (n = 8) and administered 30 min before the tests (n = 8)**p < 0.01, *p < 0.05 vs control by Student's t-test.

In order to determine the effects of PQQ and IPQ on the memory learning ability of aged mice (48 weeks old), a step-through passive avoidance test was conducted. PQQ and IPQ at doses of 5.0 mg/kg were intraperitoneally administered to 47-week-old mice (n = 8) for 7 days. As shown in Figure 5, the times taken to enter the dark room in the acquisition trials (IL trials) were almost the same in the control mice and mice administered PQQ and IPQ. However, in the regeneration trials (SIL trials) performed 24 h later, the time taken to enter the dark room was about 3-times and 2-times longer for mice administered PQQ and IPQ, respectively. From the results, it became clear that PQQ and IPQ work to improve the memory learning ability of mice.

### 3.7 MoCA test

**Figure 6:**
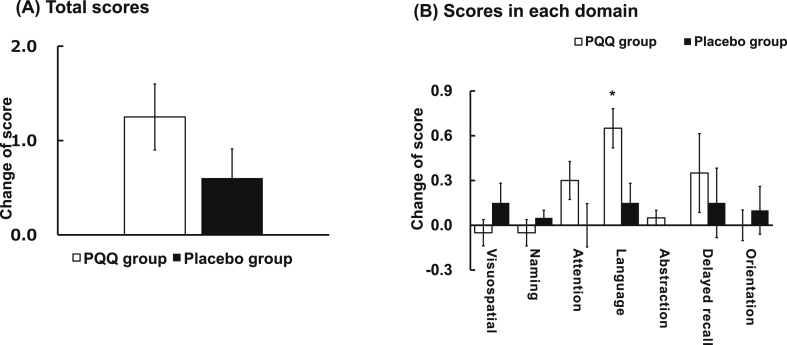
MoCA test. (A) Total scores, (B) Scores in each domain. Forty healthy men and women aged 50–71 years (14 men and 26 women) were assigned to a PQQ-containing food intake group (BioPQQ® at 20 mg per day) and a placebo food intake group. To achieve a uniform test score, each group was randomly allocated by age and gender before ingestion of the test food. The subjects were fed once daily after breakfast for 12 weeks. The MoCA test consists of 30 points (Visuospatial: 5, Naming: 3, Attention: 6, Language: 3, Abstraction: 2 Delayed recall: 5, Orientation: 6). *p < 0.05 vs control by Student's t-test.

The changes in scores were 0.60 ± 0.31 in the placebo group and 1.25 ± 0.35 in the PQQ group, indicating that PQQ ingestion increased the scores but not significantly (p = 0.118) (Figure 6). The scores were therefore analyzed for the seven domains. In the language task, the change in scores in the placebo group was 0.15 ± 0.13, while that in the PQQ group was 0.65 ± 0.13, indicating that PQQ ingestion significantly increased the scores (p < 0.05) (Figure 6).

## 4 Discussion

Various studies on PQQ have been carried out since the discovery of PQQ in 1974 [1,2,3,4]. Kasahara et al. reported PQQ as the 14th vitamin in 2003, and much interest was shown in their report. However, questions have also been raised, and more research is needed [31]. It was reported that PQQ can prevent reduction of brain function in aged persons, especially for attention and working memory [27, 30]. It was also reported that PQQ can promote lowering of blood sugar level [32, 33] and recovery from heart attack [34]. However, the mechanisms of the actions of PQQ are still unknown. In addition, because of the high reactivity of PQQ and its easy formation of adducts with amino acids, structures that actually show physiological activity *in vivo* are still unknown. In order to obtain insights into the mechanisms of the actions of PQQ and PQQH_2_ and to examine the effect of IPQ, the physiological activities of PQQ, PQQH_2_ and IPQ were compared in various experiments. As shown in Table 1, IPQ was shown to be less toxic in SK-N-SH and HepG2 cells, indicating that PQQ may exert toxicity at the quinone site or its oxidation state. The results of comparison of the effects of PQQ and IPQ on cell proliferation are shown in Figure 2A. PQQ and IPQ showed weaker effects on HepG2 cell proliferation than on SK-N-SH cell proliferation. It was thus shown that the mechanism of action might be different depending on the type of cells. There have been reports of PQQ inducing proliferation of fibroblasts and increasing NGF [12], but this report is first report on the effects of PQQ on human SK-N-SH cells and HepG2 cells. As shown in Figure 2B, PQQ had a protective effect against oxidative stress, but the protective effect of IPQ was very weak in HepG2 and SK-N-SH cells. PQQ protected against oxidative damage caused by H_2_O_2_, because it has many reducing sites, and showed a protective effect at a lower concentration than did IPQ. IPQ has fewer reducing sites due to the addition of glycine. It was suggested that HepG2 cells require more PQQ due to the greater damage caused by hydrogen peroxide than do SK-N-SH cells. This seems to be because the quinone moiety is involved in the protective effect against oxidative stress. There is a possibility that the abundance of receptors or binding proteins differs depending on cells.

6-OHDA is a neurotoxin, and it is known to cause Parkinson's disease symptoms when administered to mice. A protective effect of PQQ on 6-OHDA using human neuroblastoma SH-SY5Y cells was already reported [22]. In that study, a protective effect of PQQ on 6-OHDA was shown at a concentration of 15 μM. In the present study, SK-N-SH cell death caused by 6-OHDA was prevented by PQQ, PQQH_2_ and IPQ at concentrations of 1–100 nM. These results indicated that PQQ, PQQH_2_ and IPQ prevented neural cell death at very low concentrations. 6-OHDA has been reported to cause neuronal degeneration by inhibiting autophagy that processes denatured proteins and by inhibiting glutathione reductase [35, 36]. In SK-N-SH cells, PQQ, PQQH_2_ and IPQ are effective at low concentrations, suggesting that they are more effective in inducing autophagy than reducing oxidative stress by 6-OHDA. It was suggested that the effect of PQQ, PQQH_2_ and IPQ at a low concentration is due to their inhibitory effect on autophagy. These data also indicated that SK-N-SH might possess a more sensitive mechanism than SH-SY5Y against 6-OHDA. It was suggested that the sensitivities of PQQ binding protein or receptor in SK-N-SH cells and SH-SY5Y cells are different, but further comparison will be necessary. They might therefore be effective for prevention and treatment of Parkinson's disease. PQQ, PQQH_2_ and IPQ showed no protective effect on cell death caused by TG. The effect of PQQ and IPQ may not be related to endoplasmic reticulum stress.

In order to determine the effects of PQQ, PQQH_2_ and IPQ on the memory learning ability of older mice, a step-through type of passive avoidance test was conducted. As shown in Figure 5, in the regeneration trials (SIL trials) performed 24 h later, the time taken to enter the dark room was about 3-times and 2-times longer for mice administered PQQ and IPQ, respectively. From the results, it became clear that PQQ and IPQ work to improve memory and learning ability of mice. However, there are still many unclear points on how PQQ and IPQ are incorporated and act in the body. Further studies are needed to elucidate the mechanisms underlying the actions of PQQ and IPQ.

It was shown that PQQ and PQQH_2_ had almost same reactivities *in vivo*. It was also shown that either form or both molecular forms are active. To evaluate the effects of PQQ on human brain cognitive function, MoCA was performed. Changes in the scores were 0.60 ± 0.31 in the placebo group and 1.25 ± 0.35 in the PQQ group, indicating that PQQ ingestion increased the scores but not significantly (p = 0.118) (Figure 6A). The scores were therefore analyzed for the seven domains. In the language task, the change in scores in the placebo group was 0.15 ± 0.13, while it was 0.65 ± 0.13 in the PQQ group, indicating that PQQ ingestion significantly increased the scores (p < 0.05) (Figure 6B). MoCA is a trial that screens for mild dementia (MCI) and simple challenging tasks. Therefore, elevated scores are unlikely to occur in studies in healthy individuals. However, in our study, contrary to expectation, scores in language tasks were significantly increased. The reason for this is that the language tasks in the MoCA test are difficult. Indeed, we believe that BioPQQ® consumption significantly increased the scores in this study since the pretest scores were significantly lower than the other task scores. In human studies with BioPQQ®, touchM and Stroop tests have been shown to improve brain function [27, 29]. The present study showed that BioPQQ® is effective for improving cognitive function.

Mitchell et al. [6] reported that PQQ is highly reactive with amino acids, especially glycine, and that 98% of PQQ is converted to IPQ in vitro. They also calculated the sum of PQQ and IPQ in human breast milk was calculated to be at 140–180 ng/mL (~0.5 μM). However, the physiological activity of IPQ has not been investigated. In our biological experiment using SK-N-SH cells and in our animal experiment using mice, IPQ showed a protective effect against 6-OHDA and it promoted cell proliferation, increased COX4/1 and PGC1α, improved memory, and enhanced learning ability, as did PQQ. It was also reported that PQQ concentration in blood was increased by ingesting PQQ [37, 38]. This PQQ is not all changed to IPQ even in an environment in which PQQ is easily changed to IPQ. Considering these facts, it was suggested that the physiological effects of PQQ are the combined effects of PQQ and IPQ. It was thought that there is a converting enzyme that changes IPQ to PQQ. More experiments on the kinetics of PQQ and IPQ *in vivo* are needed. The results suggested that IPQ could be applied to supplements for cell protection and maintenance of cognition because of its low cytotoxicity and high stability.

## 5 Conclusion

It was suggested that IPQ is safer than PQQ and that the cytotoxicity of both depends on the quinone moiety. The different protective effects of PQQ and IPQ on oxidative stress were shown to be dependent on the quinone moiety. The effects of PQQ and IPQ on cell proliferation and gene expression depended on the cell type, SK-N-SH and HepG2. It is conceivable that the susceptibility differs depending on cells. Both PQQ and IPQ showed cognitive effects in mice experiments. The results indicated that IPQ functions like PQQ *in vivo*. The results also suggested that IPQ could be applied to supplements for cell protection and maintenance of cognition because of its low cytotoxicity and high stability.

## Declarations

### Author contribution statement

Yasue Yamada: Conceived and designed the experiments; Analyzed and interpreted the data; Wrote the paper.

Kazuya Nishii, Koji Kuwata, Masashi Nakamichi, Kei Nakanishi: Performed the experiments; Contributed reagents, materials, analysis tools or data.

Atsushi Sugimoto: Performed the experiments; Analyzed and interpreted the data; Contributed reagents, materials, analysis tools or data; Wrote the paper.

Kazuto Ikemoto: Contributed reagents, materials, analysis tools or data; Wrote the paper.

### Funding statement

This research is supported by the Matching Planner Program from Japan Science and Technology Agency (JST). ID;MP28116808481.

### Competing interest statement

The authors declare no conflict of interest.

### Additional information

No additional information is available for this paper.
